# Clinical profile and outcome of patients with acute kidney injury requiring dialysis—an experience from a haemodialysis unit in a developing country

**DOI:** 10.1186/s12882-016-0313-8

**Published:** 2016-07-22

**Authors:** Ahmed Ibrahim, Momina M. Ahmed, Seman Kedir, Delayehu Bekele

**Affiliations:** Moyale Hospital, Oromia Regional State, Ethiopia; Department of Internal Medicine, Nephrology Unit, Saint Paul’s Hospital Millennium Medical College, Addis Ababa, Ethiopia; Department of Public Health, Saint Paul’s Hospital Millennium Medical College, Addis Ababa, Ethiopia; Department of Obstetrics and Gynaecology, Saint Paul’s Hospital Millennium Medical College, P. O. Box 1271, Addis Ababa, Ethiopia

**Keywords:** AKI, Haemodialysis, Ethiopia

## Abstract

**Background:**

The first government funded and sustainable dialysis unit was established in Ethiopia at Saint Paul’s Hospital Millennium Medical College (SPHMMC). This has led to the development of a unique cohort of patients about which very little is known. This study was conducted to describe the clinical profile and outcome of adult Acute Kidney Injury (AKI) patients treated with intermittent haemodialysis at the dialysis center of SPHMMC.

**Methods:**

A retrospective review of clinical records of cases of AKI who required haemodialysis support during the time period from August 1, 2013 to February 1, 2015 was conducted.

**Results:**

A total of 151 cases AKI requiring dialysis were included for the study. Overall, the patients were generally younger with a mean age of 36.7 years and thus with few premorbid conditions. The most common causes of AKI were hypovolemia (22.5 %), acute glomerulonephritis (AGN) (21.9 %) and pregnancy related causes (18.5 %). Nearly a third (29.1 %) of patients succumbed to the AKI.

**Conclusion:**

Infections, AGN, obstetric causes and nephrotoxins were the primary causes of dialysis requiring AKI. Most of these causes can be prevented with simple interventions such as health education on oral rehydration, quality prenatal and emergency obstetric care, appropriate management of infections and taking appropriate precautions when prescribing potentially nephrotoxic medications.

## Background

AKI, previously known as Acute Renal Failure, is a clinical syndrome characterized by an abrupt decline in glomerular filtration rate sufficient to decrease the elimination of nitrogenous waste products (urea and creatinine) and other uremic toxins. According to a standardized definition from the Acute Kidney Injury Network proposed in 2007, AKI is a decline in kidney function during 48 h as demonstrated by an increase in serum creatinine of more than 0.3 mg/dl, an increase in serum creatinine of more than 50 % or the development of oliguria [1].

AKI is an increasingly common complication of critical illness, with some researches showing that as high as 1 in 5 adults and 1 in 3 children experiencing AKI per hospital admission. Whether occurring in the community or in the hospital, the clinical and public health importance of AKI is well established due to the association with high mortality and its separate independent effect on the risk of death and resource use [2].

The epidemiology of AKI in developing countries is unique in that certain causes, such as the infections, obstetric causes and nephrotoxins, which are largely obsolete in developed countries remain important causes. Regardless of cause, the management of AKI is mainly supportive, with dialysis being indicated when medical management fails to treat the complications. Haemodialysis is the most commonly used renal replacement therapy in Africa. However, poverty, maladministration of resources and lack of adequate funding for health in nearly all African countries has massively stunted the growth of haemodialysis as a modality of renal replacement therapy.

There had not been a sustainable government run dialysis service in Ethiopia until the establishment of the Ethio-Egyptian Haemodialysis Unit in August of 2013 at SPHMMC. This had led to the development of a unique cohort of patients about whom little is known. The aim of this study is to describe the clinical profile and outcome of all adult AKI patients treated with intermittent haemodialysis at the unit in the first 18 months since its establishment. This study is the first of its kind to describe the etiology and clinical profile of AKI patients requiring dialysis in Ethiopia.

## Methods

A hospital based retrospective cross-sectional study was done at the Ethio-Egyptian Haemodialysis Unit at SPHMMC. SPHMMC is a teaching tertiary hospital found in the Ethiopian capital Addis Ababa and gives care to patients from all corners of the country. The study population were all adult AKI patients who required haemodialysis at the unit from August 1, 2013 to February 1, 2015.

The medical record numbers of all patients dialyzed during the study period were identified from the dialysis unit log book and the clinical records retrieved from the hospital record office. All patients of age 14 and above with AKI and have been dialyzed at least once during the study period were included. In the hospital patients of age 14 and above are considered as “adults” and admitted to the adult patient wards. Those patients who had dialysis for overdose of dialyzable drugs, those who were under the age of 14 or had incomplete or missing records were excluded.

Data on socio-demographic and premorbid condition, clinical presentation and laboratory variables, dialysis variables and outcome were collected using a structured questionnaire. It was entered into and analysed using Statistical Package for Social Sciences (SPSS) Version 20.

## Results

### Socio-demographics and premorbid condition

A total of 243 patients (adult and children) underwent dialysis from August 1, 2013 to February 1, 2015. Of these, a total of 151 adult patient records were included in the study. The others were excluded because 10 were paediatric patients (under the age of 14), 9 had haemodialysis for overdose of dialyzable drugs and the rest were dialyzed for CKD or their records were missing.

The mean age of patients was 36.7 ± 14.5 years, with more than two third being in the age group between 21 to 40 years. Gender distribution was nearly equal, with 50.3 % being male. Patients came from all corners of the country with more than a third (38.4 %) coming from the capital Addis Ababa and two third (67.5 %) were urban residents (Table 1). The majority (90.7 %) of patients acquired AKI in the community.

**Table 1:** Socio-demographic characteristics of dialysis requiring AKI patients, SPHMMC, Addis Ababa, May 2015

Socio-demographic variables	Sub variable	Frequency	Percent
Gender	Male	76	50.3
	Female	75	49.7
Age(Years)	<+ 20	24	15.9
	21–30	40	26.5
	31–40	36	23.8
	41–50	27	17.9
	51–60	15	9.9
	61–70	6	4.0
	>70	3	2.0
Residence	Urban	102	67.5
	Rural	49	32.5
Region	Addis Ababa	58	38.4
	Oromiya	52	34.4
	SNNPR	19	12.6
	Amhara	15	9.9
	Harrar	3	2
	Dire Dawa	3	2
	Ethiopian Somali	1	0.7

### Premorbid condition

The most common premorbid condition identified were HIV infection (15.9 %), followed by hypertension (14.1 %) and cerebrovascular accident (3.3 %). Four patients (2.6 %) had confirmed diabetes. Only four patients had base line premorbid renal function test with mean serum creatinine value of 1.01 mg/dl.

### Causes and clinical presentation

The most common presenting features were oliguria (86.1 %), followed by edema (58.9 %), encephalopathy (49 %) and 11.3 % present with convulsion. The commonest causes of AKI were hypovolemia (22.5 %), either gastrointestinal loss (in the form of diarrhoea or vomiting) or blood loss, followed by AGN (21.9 %) and pregnancy related cause (18.5 %). Table 2 summarizes the causes of dialysis requiring AKI in the study period. Causes of hypovolemia, the most common cause of AKI in this study, were severe and uncorrected gastrointestinal loss (due to presumed infectious causes amongst others) and blood loss (due to trauma and surgery.

**Table 2:** Common Causes of dialysis requiring AKI, SPHMMC, Addis Ababa, May 2015

Common cause of dialysis requiring AKI	Number (%)	Number of deaths from a specific cause of AKI (%)
Hypovolemia	34(22.5)	10(22.7)
Pregnancy Related Cause	28(18.5)	3(6.8)
Acute Glomerulonephritis	33(21.9)	7(15.9)
Sepsis	17(11.3)	7(15.9)
Malaria	11(7.3)	3(6.8)
Post-renal/Obstructive	8(5.3)	2(4.6)
Nephrotoxic Drugs	8(5.3)	6(13.6)
Others	12(7.9)	6(13.6)
Total	151(100 %)	44(100 %)

Though difficult to accurately diagnose in our setup due to lack of availability of renal biopsy, AGN (diagnosed through clinical means) was found to be a common cause of AKI requiring dialysis. We diagnosed AGN clinically when there is an acute onset of oliguria followed by body swelling, with new onset hypertension, glomerular haematuria and some degree of proteinuria.

The pregnancy related causes include severe preeclampsia/eclampsia in 20(12.5 %), puerperal sepsis in 5(3.3 %), postpartum haemorrhage in 2(1.3 %) and one case (0.7 %) of septic abortion.

The most common nephrotoxins were the aminoglycoside gentamycin and the antiretroviral drug Tenofovir, followed by antineoplastic drugs and vancomycin. The common post renal obstructive causes were stone disease, followed by benign prostatic hyperplasia and strictures. Other causes accounting for a minority of cases include malignant hypertension, Tumor Lysis Syndrome, rhabdomyolysis following electric burn injury and interstitial nephritis.

Twenty Seven (16.9 %) of patients had AKI superimposed on CKD, with the most common cause of CKD being hypertension (51.9 %). A majority of these patients had not been diagnosed initially with CKD as they were presenting to a health facility for the first time. Diabetes and hypertension accounted for a combined 56 % of causes of these background CKD and a quarter (22 %) having no immediately identifiable cause.

### Laboratory values

Nearly all of the patients had mild anaemia on admission, and had an average drop of 2 g/dl of haemoglobin at discharge. The mean levels of creatinine at admission and discharge were 10.18 ± 5.19 and 4.97 ± 3.54 mg/dl respectively and for potassium the value was 5.03 ± 1.35 and 4.06 ± 1.02 mEq/L at admission and discharge respectively. Table 3 summarizes the values for selected laboratory parameters.

**Table 3:** Selected Laboratory Values dialysis requiring AKI patients, SPHMMC, Addis Ababa, May 2015

Lab values	Point in time	Mean ± standard deviation
Urea	Admission	218.34 ± 134.53 mg/dl
	Discharge	104.25 ± 87.72 mg/dl
Creatinine	Admission	10.18 ± 5.19 mg/dl
	Discharge	4.97 ± 3.54 mg/dl
Serum K	Admission	5.03 ± 1.35 mEq/L
	Discharge	4.06 ± 1.02 mEq/L
WBC count	Admission	12.56 ± 8.03 × 10^3^/ml
	Discharge	9.03 ± 5.35 × 10^3^/ml
Haemoglobin	Admission	10.06 ± 2.94gm/dl
	Discharge	8.85 ± 2.92 gm/dl
Platelet count	Admission	254.1 ± 180.57 × 10^3^/ml
	Discharge	278.82 ± 179.06 × 10^3^/ml

### Dialysis and outcome variables

The average number of dialysis sessions was 4.8, with a maximum of 31 and a minimum of 1. The most common indication for dialysis was refractory fluid overload (89.4 %), followed by uremic signs and symptoms (61.1 %) (Table 4).

**Table 4:** Indication for dialysis of AKI patients, SPHMMC, Addis Ababa, May 2015

Dialysis indication^a^	Frequency (Percent)	
	Yes	No
Refractory Fluid Overload	135 (89.4 %)	16 (10.6 %)
Uremic signs and symptoms	93 (61.6 %)	58 (38.4 %)
Hyperkalemia	44 (29.1 %)	107 (70.9 %)
Metabolic Acidosis	14 (9.3 %)	137 (90.7 %)

^a^Sum is more than 100 % as most patients had more than one indication for dialysis

Average duration of hospital stay was 20.7 days, with range of 1–92 days. Of the 151 patients, 80 (53 %) were discharged improved, and 44 (29.1 %) died, with the remaining absconding (10.6 %) or progressing to end stage renal disease (7.3 %). Hypovolemia, AGN and sepsis are the causes of AKI which accounted for more than half (54.5 %) of deaths (Table 2). The most common cause of death was sudden cardiac arrest and the causes of death for the others are shown in Table 5.

**Table 5:** Cause of death of dialysis requiring AKI patients, SPHMMC, Addis Ababa, May 2015

Cause of death	Frequency	Percent
Sudden Cardiac Death	18	42.9 %
Septic Shock	14	33.3 %
ARDS	8	19 %
Cardiogenic Shock	1	2.4 %
Others	1	2.4 %
Total	44	100 %

## Discussion

As the first study on dialysis requiring AKI, the study has shade some light on the epidemiology, causes and prognosis for these patients in the Ethiopian context. The relatively young age of patients is consistent with results from other parts of Africa and can be explained by the predominantly young overall population demographics of most sub-Saharan countries from which similar researches were done [3, 4].

The prevalence of premorbid conditions is relatively low which might be explained by the relatively younger and thus healthier condition of the patients. HIV infection is the most common premorbid condition identified. This is a reflection of the overall prevalence of HIV infection in the country which is 1.5 % of the 15–49 age group according to the 2011 Ethiopian demographic health survey [5]. Previous studies has shown that patients with HIV infection are more likely to develop kidney disease because of the HIV virus itself, the opportunistic infections arising from the immune-deficient state and the drugs that these patients take [6]. In line with results from multiple studies, diabetes and hypertension accounted for a the majority of cases of background CKDs [4].

The most common presenting features were oliguria followed by edema and encephalopathy and this is similar to findings from Emen-Chioma from Nigeria and Bagshaw from Calgary, Alberta Canada [3, 7].

In contrast to developed countries infections, obstetric complications and nephrotoxins are the major cause of AKI in the tropics. This is also reflected in this study, where the top 5 causes fall into one of these 3 categories; hypovolemia, either gastrointestinal loss (in the form of diarrhoea or vomiting) or blood loss, followed by AGN and pregnancy related causes. Similar results were found in studies done in other parts of Africa, where hypovolemia (or one of its causes) ranks in the top 3 causes of AKI [4, 8–11]. Though initially correctable with volume correction, as cellular dysfunction continues, renal tubular cells sustain ischemic injury which may persist after correction of the initial hypoperfusion [3].

AGN was found to be a common cause of AKI requiring dialysis and this corresponds to previous reports that AGN appears to be higher in developing countries, as evidenced by the high burden in countries like Turkey and Northern Africa [12].

All the pregnancy related causes which includes severe preeclampsia/eclampsia, puerperal sepsis in, postpartum haemorrhage and septic abortion are preventable. This indicates the need for increasing access to appropriate and good quality prenatal and emergency obstetric care services.

The average number of dialysis sessions was 4.8, which is nearly twice that of what was given at a center in Nigeria [3]. However, in the Nigerian study they have stated that the reason for the relatively smaller number of dialysis sessions is financial, as most of their patients could not afford to keep paying the cost and abscond after a couple of cycles.

The most common indication for dialysis was refractory fluid overload and uremic signs and symptoms. These results are in contrast to those from an ICU center in Morocco and that of three health boards in Scotland, which has found the most common indications to be refractory hyperkalemia, followed by metabolic acidosis [8, 9]. One possible explanation may be the difficulty in accurately diagnosing metabolic acidosis due to lack of blood gas analysis in the Ethiopian setup.

The mortality rate of 29.1 % in our study is much lower than the data from a global meta-analysis of studies done across the globe which has shown a pooled AKI-associated mortality rate 49.4 % for dialysis requiring AKI [2]. However, research from other parts of the world has shown that in AKI, a stable survival rate is not achieved until after 30–60 days postrecovery [13]. Thus considering the fact that this study did not assess survival post discharge, it is difficult to predict that all of the 80 patients who were discharged improved will have good long term prognosis. Furthermore, the fate of 10 % of patients is unknown as they absconded and discharged against medical advice.

## Conclusion

Consistent with other studies from developing world, this study has also shown that infections, AGN, obstetric causes and nephrotoxins are the primary causes of dialysis requiring AKI at Saint Paul’s hospital. Most of these causes can be prevented with simple interventions such as health education on oral rehydration, quality prenatal and emergency obstetric care, appropriate management of infections and taking appropriate precautions when prescribing potentially nephrotoxic medications.

Future studies may also benefit by better identifying modifiable risk factors to prevent the development of AKI from the outset. Patients came from all corners of the country and expansion of acute dialysis services and subspecialty nephrology care to other parts of the country is necessary.

## Abbreviations

AGN, Acute Glomerulonephritis; AKI, Acute Kidney Injury; ARDS, Adult Respiratory Distress Syndrome; CKD, Chronic Kidney Disease; SNNPR, Sothern Nations Nationalities People Region; SPHMMC, Saint Paul’s Hospital Millennium Medical College; WBC, White Blood Cell
